# Reconciled multiplicative relational two-stage network data envelopment analysis

**DOI:** 10.1016/j.mex.2025.103758

**Published:** 2025-12-11

**Authors:** M. Burak Erturan

**Affiliations:** Dr.,Head Supply Engineer, General Directorate of State Hydraulic Works, Directorate of Region 13, Barış Mah. Halide Edip Cad. Kepez Antalya, Turkiye

**Keywords:** Data envelopment analysis, Two-stage network DEA, Data reconciliation, Relational, Multiplicative, Efficiency

## Abstract

Relational two-stage network DEA (data envelopment analysis) is an approach for two-stage systems, where all input, output and the intermediate products have same weights regardless of which process they are related. One of the main approaches to relational model is the multiplicative relational approach where main process efficiency is the product of two sub-process efficiencies. A shortcoming of the relational model is that the solution of the sub-process efficiencies may not be unique. Researchers have developed some models to overcome this problem, like prioritizing one sub-process to the other or assessing the sub-process efficiencies first and then the main process efficiency. In this study, a novel methodology is presented for a fairer efficiency assessment, where none of the processes is prioritized.

• Reconciled Multiplicative Relational (RMR) model presented in this study uses data reconciliation method to determine maximum efficiency values for all processes simultaneously within the relational constraints.
• RMR model has unique efficiency assessment solution, which solves the non-uniqueness problem and useful where none of the DMUs are preferred to another or there is no previous information in that matter.

## Graphical abstract

**Figure:**
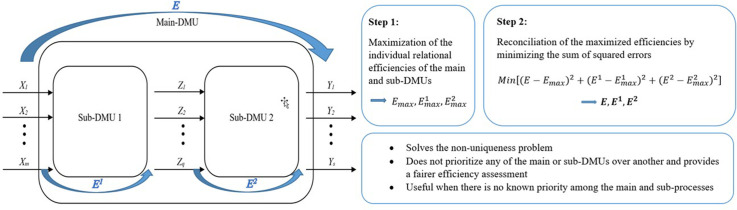
Graphical Abstract.

**Specifications table**

**Table:** 

**Subject area**	Mathematics and Statistics
**More specific subject area**	Network Data Envelopment Analysis
**Name of your method**	Reconciled Multiplicative Relational Two-Stage Network Data Envelopment Analysis
**Name and reference of original method**	Multiplicative Relational Two-Stage Network DEA C. Kao, S.N. Hwang, Efficiency decomposition in two-stage data envelopment analysis: An application to non-life insurance companies in Taiwan, Eur. J. Oper. Res. 185 (2008) 418–429. https://doi.org/10.1016/j.ejor.2006.11.041
**Resource availability**	Data is presented within the study

## Background

The multiplicative relational (MR) two-stage network data envelopment analysis (NDEA) model presented in [1], assumes the main decision-making unit (DMU) efficiency is the product of two sub-stage efficiencies. The weights of the intermediate products are the same for both the first and the second stages. A shortcoming of the multiplicative relational model is that the decomposition of the main-process efficiency to the sub-process efficiencies may be not unique [1]. There are different approaches to solve this problem. Kao and Hwang [1] proposed a solution with a post-optimization process by prioritizing one stage to the other. First, the main-process efficiency is maximized, and then the preferred stage efficiency is maximized by keeping the main-process efficiency constant at its maximum. Liu [2] proposed a method to combine these two stages in one-step. Zhou et al. [3] uses a Nash bargaining game model to determine an efficiency decomposition for the sub-processes while keeping the main-process efficiency unchanged. The model proposed in [4] determines the main and sub-process efficiencies by using DMU-specific degree of priorities. In the compositional model of An et al. [5], first the individual non-relational (independent) efficiencies of sub-processes are determined. Next, the ratio of independent efficiency scores of the sub-processes are found, and then the decomposition of the overall efficiency is done using this ratio. Lu and Cheng [6] proposed two alternative secondary goals: maximizing the total efficiencies of sub-processes and maximizing the individual sub-efficiencies simultaneously.

In this study, a novel reconciled multiplicative relational (RMR) two-stage network data envelopment analysis is presented that uses data reconciliation method for efficiency assessment. Data reconciliation is a process usually employed in experimental sciences or industry, where measured variable values are not met with the theoretic specific consistencies. Noised or biased measuring instruments or disturbances of different nature usually affect the measurements. Therefore, the measured values may not be consistent with related physical laws. In such cases, measured values are adjusted such that sum of squared errors (differences between measured and reconciled final values) are minimized and adjusted values become consistent with related physics laws [7,8].

Proposed model consists of two main steps. In the first step, maximum individual efficiencies of main and sub-processes are determined within the relational constraints. These values are maximum possible individual efficiencies of DMUs within the relational production possibility set. In the second step, a data reconciliation process is employed such that all relational (multiplicative) model constraints are satisfied and the reconciled efficiency values are as close as possible to their maximum values found in the first step. The main difference of the proposed model from others is that this model does not prioritize main-DMU or sub-DMUs, but treat them as equally important variables. Therefore, a fairer efficiency assessment is achieved. Different models in the literature give the user/manager the opportunity to determine the efficiencies according to his/her preferences. Presented model is more suitable for the situations where there is no preference towards the importance of main or sub-processes, or no previous information about that matter.

Presented RMR model differs from the previous models with its ability to solve the non-uniqueness problem with fairer assessment of the efficiencies. All previous models prioritize the main-DMU efficiency over the sub-DMU efficiencies. It means that, maximum possible value of the main-DMU efficiency is found first, and does not change during the process. The non-uniqueness problem occurs during the efficiency assessment of sub-DMUs, because mostly there is no unique assessment of those efficiencies. Models in [1,2] and [4] solves the problem by adding another prioritization between sub-DMUs, which is pre-determined by the user/manager. In [5], sub-DMU efficiencies are determined according to the ratio of their independent efficiencies, while in [3] the assessment is done by a bargaining game between sub-DMUs. In the model of [6], stage efficiencies and their sum is maximized simultaneously. Presented RMR model is different from aforementioned models such that none of the main or sub-DMUs has priority over another. Unlike other models, main-DMU efficiency can be change during the reconciliation process and there is no non-uniqueness problem of efficiency assessment.

## Method details

### Multiplicative relational (MR) two-stage network DEA

**Fig. 1:**
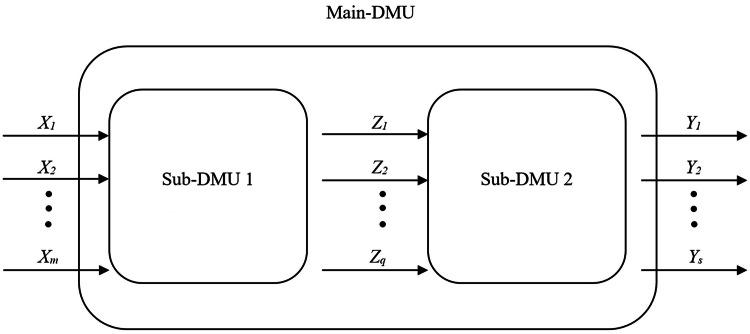
Representation of a two-stage production system.

In the study of Kao and Hwang [1], the multiplicative relational two-stage system is proposed. In Fig. 1, a two-stage production system is demonstrated. The main process of *k*th DMU uses *m* inputs *X_ik_* (*i* *=* *1,2,…,m*) to produce *s* outputs *Y_rk_* (*r* *=* *1,2,…,s*). In addition, there are *q* intermediate products *Z_pk_* (*p* *=* *1,2,…,q*), which are the outputs of the first stage and the inputs of the second one. Therefore, the linear program in Model (1) can determine the efficiency of main-process of *k*th DMU among n compared processes.

(1) $$\begin{array}{c} E_{k}=\max\sum_{r=1}^{s}u_{r}Y_{rk}\\ s.t.\\ \sum_{i=1}^{m}v_{i}X_{ik}=1,\\ \sum_{r=1}^{s}u_{r}Y_{rj}-\sum_{i=1}^{m}v_{i}X_{ij}\leq 0,\;\;\;\;\;j=1,\ldots ,n,\\ \sum_{p=1}^{q}w_{p}Z_{pj}-\sum_{i=1}^{m}v_{i}X_{ij}\leq 0,\;\;\;\;\;j=1,\ldots ,n,\\ \sum_{r=1}^{s}u_{r}Y_{rj}-\sum_{p=1}^{q}w_{p}Z_{pj}\leq 0,\;\;\;\;\;j=1,\ldots ,n,\\ u_{r},v_{i},w_{p}\geq \varepsilon ,\;\;\;\;\;r=1,\ldots ,s,\;\;\;\;\;i=1,\ldots ,m,\;\;\;\;\;p=1,\ldots ,q. \end{array}$$

where, *ε* is a small non-Archimedean number.

The weights and therefore the weighted values of the intermediate products (*w_p_, Z_pj_*) are same for stages 1 and 2. After finding the optimal weight values u_r_*, v_i_*, and w_p_*, main (*E_k_*) and sub-process (*E_k_^1^, E_k_^2^*) efficiencies can be found as:

(2) $$\begin{array}{c} E_{k}=E_{k,max}=\sum_{r=1}^{s}u_{r}^{*}Y_{rk},\\ E_{k}^{1}=\frac{\sum_{p=1}^{q}w_{p}^{*}Z_{pk}}{\sum_{i=1}^{m}v_{i}^{*}X_{ik}},\\ E_{k}^{2}=\frac{\sum_{r=1}^{s}u_{r}^{*}Y_{rk}}{\sum_{p=1}^{q}w_{p}^{*}Z_{pk}}, \end{array}$$

Therefore, the efficiency assessment is multiplicative as *E_k_ = E_k_^1^ x E_k_^2^*. On the other hand, the decomposition of *E_k_^1^* and *E_k_^2^* might not be unique, and one solution for this problem is maximizing one of the sub-DMU efficiencies while keeping the main-DMU efficiency at its maximum found in model (1). If the efficiency of stage 1 (*E_k_^1^*) is desired to be maximized, then the problem could be formulated as model (3) with the main process efficiency value *E_k_* calculated from Model (1).

(3) $$\begin{array}{c} E_{k}^{1}=\max\sum_{p=1}^{q}w_{p}Z_{pk}\\ s.t.\\ \sum_{i=1}^{m}v_{i}X_{ik}=1,\\ \sum_{r=1}^{s}u_{r}Y_{rk}-E_{k}\sum_{i=1}^{m}v_{i}X_{ik}=0,\\ \sum_{r=1}^{s}u_{r}Y_{rj}-\sum_{i=1}^{m}v_{i}X_{ij}\leq 0,\;\;\;\;\;j=1,\ldots ,n,\\ \sum_{p=1}^{q}w_{p}Z_{pj}-\sum_{i=1}^{m}v_{i}X_{ij}\leq 0,\;\;\;\;\;j=1,\ldots ,n,\\ \sum_{r=1}^{s}u_{r}Y_{rj}-\sum_{p=1}^{q}w_{p}Z_{pj}\leq 0,\;\;\;\;\;j=1,\ldots ,n,\\ u_{r},v_{i},w_{p}\geq \varepsilon ,\;\;\;\;\;r=1,\ldots ,s,\;\;\;\;\;i=1,\ldots ,m,\;\;\;\;\;p=1,\ldots ,q. \end{array}$$

After the first stage efficiency (*E_k_^1^=E_k,max_^1^*) is calculated, *E_k_^2^* is determined from *E_k_^2^* = *E_k,_*_max_ / *E_k,max_^1^*.

### Reconciled multiplicative relational (RMR) two-stage network DEA

The non-uniqueness problem in multiplicative relational two-stage network DEA model has led researchers to develop various and mostly two-step models. Aforementioned multiplicative relational models [[1], [2], [3], [4], [5], [6]] solve the non-uniqueness problem but prioritizes main or sub-efficiencies over others. For example, in the original model [1] the main DMU efficiency is maximized first, then keeping that value constant one of the sub-DMU efficiency is maximized. These models are useful when main or sub-DMUs have differences in importance. On the other hand, there might be situations where there is not any known priorities among main or sub-DMUs, and a fairer efficiency assessment model needed.

In this study, reconciled relational multiplicative two-stage network DEA model is presented. The efficiency assessment of the model is based on data reconciliation process used especially in experimental sciences. Data reconciliation is applied when measured variables are not consistent with related physics laws. In such cases, measured values are updated (reconciled) such that the sum of squared changes of variables are minimized while maintaining the consistencies according to related physics laws.

The model presented consists of two steps. In the first step, individual maximum efficiency values of main (*E_k,_*_max_) and sub-DMUs (*E_k,max_^1^, E_k,max_^2^*) are found considering the relational constraints. Thus, the following linear programs (4–6) are solved separately:

(4) $$\begin{array}{c} E_{k,max}=\max\sum_{r=1}^{s}u_{r}Y_{rk}\\ s.t.\\ \sum_{i=1}^{m}v_{i}X_{ik}=1,\\ \sum_{r=1}^{s}u_{r}Y_{rj}-\sum_{i=1}^{m}v_{i}X_{ij}\leq 0,\;\;\;\;\;j=1,\ldots ,n,\\ \sum_{p=1}^{q}w_{p}Z_{pj}-\sum_{i=1}^{m}v_{i}X_{ij}\leq 0,\;\;\;\;\;j=1,\ldots ,n,\\ \sum_{r=1}^{s}u_{r}Y_{rj}-\sum_{p=1}^{q}w_{p}Z_{pj}\leq 0,\;\;\;\;\;j=1,\ldots ,n,\\ u_{r},v_{i},w_{p}\geq \varepsilon ,\;\;\;\;\;r=1,\ldots ,s,\;\;\;\;\;i=1,\ldots ,m,\;\;\;\;\;p=1,\ldots ,q. \end{array}$$

(5) $$\begin{array}{c} E_{k,max}^{1}=\max\sum_{p=1}^{q}w_{p}Z_{pk}\\ s.t.\\ \sum_{i=1}^{m}v_{i}X_{ik}=1,\\ \sum_{r=1}^{s}u_{r}Y_{rj}-\sum_{i=1}^{m}v_{i}X_{ij}\leq 0,\;\;\;\;\;j=1,\ldots ,n,\\ \sum_{p=1}^{q}w_{p}Z_{pj}-\sum_{i=1}^{m}v_{i}X_{ij}\leq 0,\;\;\;\;\;j=1,\ldots ,n,\\ \sum_{r=1}^{s}u_{r}Y_{rj}-\sum_{p=1}^{q}w_{p}Z_{pj}\leq 0,\;\;\;\;\;j=1,\ldots ,n,\\ u_{r},v_{i},w_{p}\geq \varepsilon ,\;\;\;\;\;r=1,\ldots ,s,\;\;\;\;\;i=1,\ldots ,m,\;\;\;\;\;p=1,\ldots ,q. \end{array}$$

(6) $$\begin{array}{c} E_{k,max}^{2}=\max\sum_{r=1}^{s}u_{r}Y_{rk}\\ s.t.\\ \sum_{p=1}^{q}w_{p}Z_{pk}=1,\\ \sum_{r=1}^{s}u_{r}Y_{rj}-\sum_{i=1}^{m}v_{i}X_{ij}\leq 0,\;\;\;\;\;j=1,\ldots ,n,\\ \sum_{p=1}^{q}w_{p}Z_{pj}-\sum_{i=1}^{m}v_{i}X_{ij}\leq 0,\;\;\;\;\;j=1,\ldots ,n,\\ \sum_{r=1}^{s}u_{r}Y_{rj}-\sum_{p=1}^{q}w_{p}Z_{pj}\leq 0,\;\;\;\;\;j=1,\ldots ,n,\\ u_{r},v_{i},w_{p}\geq \varepsilon ,\;\;\;\;\;r=1,\ldots ,s,\;\;\;\;\;i=1,\ldots ,m,\;\;\;\;\;p=1,\ldots ,q. \end{array}$$

*E_k,_*_max_, *E_k,max_^1^, E_k,max_^2^* are found by solving models (4–6). The values found usually do not maintain the multiplicative consistency of *E_k,_*_max_ *= E_k,max_^1^ x E_k,max_^2^*. Note that, in the original method [1], only the efficiency of main-DMU is maximized (model 1), which is exactly the same of model (4). Different from the original MR method, in RMR methodology stage 1 and stage 2 efficiencies are also maximized (models 5–6) separately.

In the second step of RMR method, a data reconciliation process is applied using the maximum efficiency values of DMUs found in (4–6). If the reconciled final values of the efficiencies are *E_k_, E_k_^1^* and *E_k_^2^*, then the optimization problem is:

(7) $$\begin{array}{c} R=\min\left[{\left(E_{k,max}-E_{k}\right)}^{2}+{\left(E_{k,max}^{1}-E_{k}^{1}\right)}^{2}+{\left(E_{k,max}^{2}-E_{k}^{2}\right)}^{2}\right]\\ s.t.\\ \sum_{r=1}^{s}u_{r}Y_{rj}-\sum_{i=1}^{m}v_{i}X_{ij}\leq 0,\;\;\;\;\;j=1,\ldots ,n,\\ \sum_{p=1}^{q}w_{p}Z_{pj}-\sum_{i=1}^{m}v_{i}X_{ij}\leq 0,\;\;\;\;\;j=1,\ldots ,n,\\ \sum_{r=1}^{s}u_{r}Y_{rj}-\sum_{p=1}^{q}w_{p}Z_{pj}\leq 0,\;\;\;\;\;j=1,\ldots ,n,\\ E_{k}=\sum_{r=1}^{s}u_{r}Y_{rk},\\ E_{k}^{1}=\frac{\sum_{p=1}^{q}w_{p}Z_{pk}}{\sum_{i=1}^{m}v_{i}X_{ik}}\;,\\ E_{k}^{2}=\frac{\sum_{r=1}^{s}u_{r}Y_{rk}}{\sum_{p=1}^{q}w_{p}Z_{pk}}\;,\\ u_{r},v_{i},w_{p}\geq \varepsilon ,\;\;\;\;\;r=1,\ldots ,s,\;\;\;\;\;i=1,\ldots ,m,\;\;\;\;\;p=1,\ldots ,q. \end{array}$$

Note that model (7) is a quadratic programming problem with linear constraints, and the variables are final reconciled main and sub-DMU efficiency values (*E_k_, E_k_^1^* and *E_k_^2^*). Therefore, the quadratic objective in (7) may require nonlinear solvers, and the software used should be selected accordingly. In this study, the applications in “method validation” are performed by MS Excel Solver add-in with GRG Nonlinear engine, efficiently.

**Fig. 2:**
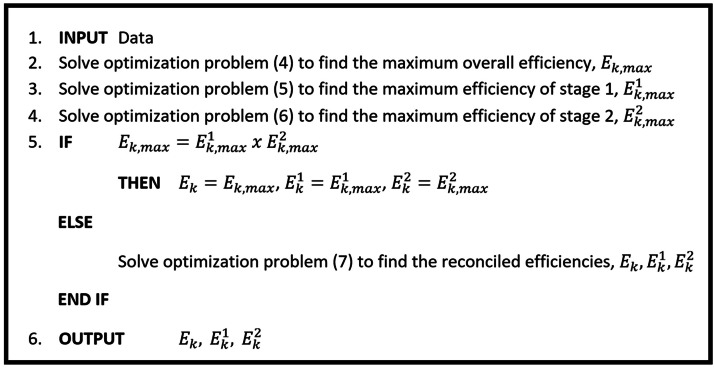
A pseudocode representation of reconciled multiplicative relational model.

A representation of proposed model for *k*th process is presented as a pseudocode in Fig. 2. Therefore, the two-step process of the RMR model can be written as: Inputs → Step 1: solve models (4)-(6) → Step 2: solve reconciliation model (7) → Outputs

### Uniqueness of the efficiency assessment

A fundamental principle of DEA is that the efficiency is strictly positive [9,10]. As zero efficiency would imply an infinite return to scale or complete irrelevance of all inputs and outputs [11], a small positive non-Archimedean number (*ε*) is used as a lower bound for model weights. This infinitesimal number ensures that no input or output factor is entirely disregarded by assigning a zero multiplier, and prevents DMU from not just zero efficiency, but also the weak efficiency problem [12,13]. The weak efficiency problem occurs when a DMU is efficient (*E* = 1) and positioned on the efficiency frontier, but it is possible to reduce at least one input or increase at least one output without affecting other inputs or outputs. Which means that, while the efficiency of DMU lies on the efficient frontier, it is not operating at its full potential, as some inputs could still be decreased or outputs increased [14]. The appropriate value of the non-Archimedean number is important. Because, too high values can lead infeasible models while too low ones might fail to adequately distinguish between efficient and weakly efficient units. While, various studies have sought to establish practical bounds and methods for determining an appropriate ε value, most researchers often set to 10^–6^ or 10^–7^ practically [9,15]. Therefore, by using a non-Archimedean number in the model (stated in model 1), all main and sub-DMUs are strictly positive.

As stated earlier, the main problem with conventional multiplicative relational two-stage NDEA model is the non-uniqueness of the efficiency assessment. Method presented in this paper can be checked for the uniqueness of the solution. The optimization model (7) proposed could be written in terms of efficiency values. The objective function is:

$$f\left(E_{k}^{1},E_{k}^{2}\right)=\;\left[{\left(E_{k,\max}-\left(E_{k}^{1}E_{k}^{2}\right)\right)}^{2}+{\left(E_{k,\max}^{1}-E_{k}^{1}\right)}^{2}+{\left(E_{k,\max}^{2}-E_{k}^{2}\right)}^{2}\right]$$

where,

(8) $$\begin{array}{c} 0<E_{k,min}^{1}\leq E_{k}^{1}\leq E_{k,max}^{1}\leq 1\\ 0<E_{k,min}^{2}\leq E_{k}^{2}\leq E_{k,max}^{2}\leq 1\\ 0<E_{k,min}\leq E_{k}^{1}xE_{k}^{2}\leq E_{k,max}\leq 1\;\;\;\;\;\;\;\;\;\left(E_{k}=E_{k}^{1}xE_{k}^{2}\right) \end{array}$$

*E_k,max_^1^, E_k,max_^2^, E_k,_*_max_, *E_k,min_^1^, E_k,min_^2^*, and *E_k,_*_min_ are relational maximum and minimum efficiency values. *E_k,_*_max_, *E_k,max_^1^* and *E_k,max_^2^* are already found in models (4–6), whereas *E_k,_*_min_, *E_k,min_^1^* and *E_k,min_^2^* can be found similarly by minimization.

If a function’s Hessian matrix’s leading principal minors are positive on the related domain, this function is called definite positive. A definite positive function is strictly convex, which means it has only one global minima. Therefore, minimization of a strictly convex function results with a unique solution of the aforementioned global minima within its domain [16,17]. The Hessian matrix (*H*) and its leading principle minors can be checked for the function in model (8). The Hessian matrix is:

(9) $$H=\left[\begin{array}{cc} \frac{\partial^{2}f\left(E_{k}^{1},E_{k}^{2}\right)}{\partial {\left(E_{k}^{1}\right)}^{2}} & \frac{\partial^{2}f\left(E_{k}^{1},E_{k}^{2}\right)}{\partial E_{k}^{1}\partial E_{k}^{2}}\\ \frac{\partial^{2}f\left(E_{k}^{1},E_{k}^{2}\right)}{\partial E_{k}^{2}\partial E_{k}^{1}} & \frac{\partial^{2}f\left(E_{k}^{1},E_{k}^{2}\right)}{\partial {\left(E_{k}^{2}\right)}^{2}} \end{array}\right]=\left[\begin{array}{cc} 2{\left(E_{k}^{2}\right)}^{2}+2 & 4E_{k}^{1}E_{k}^{2}-2E_{k,max}\\ 4E_{k}^{1}E_{k}^{2}-2E_{k,max} & 2{\left(E_{k}^{1}\right)}^{2}+2 \end{array}\right]$$

Then, the leading principle minors are,

(10) $$D_{1}=\frac{\partial^{2}f\left(E_{k}^{1},E_{k}^{2}\right)}{\partial {\left(E_{k}^{1}\right)}^{2}}=2{\left(E_{k}^{2}\right)}^{2}+2$$

(11) $$D_{2}=\left(\frac{\partial^{2}f\left(E_{k}^{1},E_{k}^{2}\right)}{\partial {\left(E_{k}^{1}\right)}^{2}}.\frac{\partial^{2}f\left(E_{k}^{1},E_{k}^{2}\right)}{\partial {\left(E_{k}^{2}\right)}^{2}}\right)-\left(\frac{\partial^{2}f\left(E_{k}^{1},E_{k}^{2}\right)}{\partial E_{k}^{1}\partial E_{k}^{2}}.\frac{\partial^{2}f\left(E_{k}^{1},E_{k}^{2}\right)}{\partial E_{k}^{2}\partial E_{k}^{1}}\right)=\left(\left(2{\left(E_{k}^{2}\right)}^{2}+2\right).\left(2{\left(E_{k}^{1}\right)}^{2}+2\right)\right)-{\left(4E_{k}^{1}E_{k}^{2}-2E_{k,max}\right)}^{2}$$

Since *E_k_^2^>0*,

(12) $$D_{1}=\left(2{\left(E_{k}^{2}\right)}^{2}+2\right)>2$$

Furthermore, for the second principle minor (*D_2_*), while *E_k_^1^>0, E_k_^1^ x E_k_^2^≤ E_k,max_≤1*,

(13) $$\left(\left(2{\left(E_{k}^{2}\right)}^{2}+2\right).\left(2{\left(E_{k}^{1}\right)}^{2}+2\right)\right)>4$$

(14) $${\left(4E_{k}^{1}E_{k}^{2}-2E_{k,max}\right)}^{2}\leq 4.$$

Therefore,

(15) $$D_{2}=\left(\left(2{\left(E_{k}^{2}\right)}^{2}+2\right).\left(2{\left(E_{k}^{1}\right)}^{2}+2\right)\right)-{\left(4E_{k}^{1}E_{k}^{2}-2E_{k,max}\right)}^{2}>0$$

Hence, the Hessian matrix’s (*H*) leading principle minors (*D_1_, D_2_*) are positive, the objective function is strictly convex and our proposed model has a unique solution.

### Efficiency considerations

The main purposes of applying data envelopment analysis are identifying the efficient (*E* *=* *1*) and inefficient (*0* *<* *E* *<* *1*) nodes and ranking them according to their efficiency values. If the *k*th main process is efficient according to conventional MR model of [1], hence the equation *E_k_ = E_k_^1^= E_k_^2^=1* is satisfied. Then, it is also efficient according to reconciled relational model since the efficiency values are at their maximum and multiplicatively-consistent. In that circumstance, the reconciliation step is unnecessary and gives the same result even it is applied. On the other hand, any efficient sub-DMU (*E_k_^1^=1* or *E_k_^2^=1*) according to classical MR model, or after the first step (*E_k,max_^1^=1* or *E_k,max_^2^=1*) of RMR model; it is not necessarily efficient after the second step.

## Method validation

### Taiwanese non-life insurance companies

In the study of Kao and Hwang [1] the relational model presented is applied to 24 Taiwanese non-life insurance companies. This dataset has been one of the benchmark datasets for two-stage network DEA efficiency analysis. Several researchers use this dataset for application and comparison purposes, such as [[18], [19], [20], [21]]. Similarly, in this study the dataset of 24 Taiwanese non-life insurance companies [1] is used for application of the proposed methodology and compared with independent [22] and relational multiplicative model with sub-stage prioritizing [1].

The non-life insurance industry provides services to generate profit. However, the profit is not earned just from insurance, but also from investment. Non-life insurance industry uses the insurance premiums as capital for investment. The main process of non-life insurance companies can be divided into two as marketing and investment processes [1]. In the first sub-process, inputs are operation and insurance expenses, where the outputs are direct written and reinsurance premiums. In this stage, direct written premiums and reinsurance premiums are received from clients and other insurance companies respectively. In the second stage, the inputs are direct written and reinsurance premiums while underwriting and investment profits are the outputs. The second sub-process is the investment stage, where premiums are invested in a portfolio to earn profit [1].

Dataset presented in Table 1 consists of 24 non-life insurance companies in Taiwan and the values are the averages of the years 2001 and 2002. Inputs and outputs used are as follows [1]: Operation expenses (X1) are the salaries of the employees and various costs incurred in daily operation. Insurance expenses (X2) are the expenses of agencies, brokers etc.; and others associated with marketing. Underwriting profit (Y1) is the profit from the insurance business, while investment profit (Y2) is the profit from the investment portfolio. Direct written premiums (Z1) are the premiums received from clients, while reinsurance premiums (Z2) are received from ceding companies.

**Table 1:** Dataset for 24 Taiwanese non-life insurance companies [1].

**Company**	**X1**	**X2**	**Z1**	**Z2**	**Y1**	**Y2**
**Taiwan Fire**	1178,744.0	673,512.0	7451,757.0	856,735.0	984,143.0	681,687.0
**Chung Kuo**	1381,822.0	1352,755.0	10,020,274.0	1812,894.0	1228,502.0	834,754.0
**TaiPing**	1177,494.0	592,790.0	4776,548.0	560,244.0	293,613.0	658,428.0
**China Mariners**	601,320.0	594,259.0	3174,851.0	371,863.0	248,709.0	177,331.0
**Fubon**	6699,063.0	3531,614.0	37,392,862.0	1753,794.0	7851,229.0	3925,272.0
**Zurich**	2627,707.0	668,363.0	9747,908.0	952,326.0	1713,598.0	415,058.0
**Taian**	1942,833.0	1443,100.0	10,685,457.0	643,412.0	2239,593.0	439,039.0
**Ming Tai**	3789,001.0	1873,530.0	17,267,266.0	1134,600.0	3899,530.0	622,868.0
**Central**	1567,746.0	950,432.0	11,473,162.0	546,337.0	1043,778.0	264,098.0
**The First**	1303,249.0	1298,470.0	8210,389.0	504,528.0	1697,941.0	554,806.0
**Kuo Hua**	1962,448.0	672,414.0	7222,378.0	643,178.0	1486,014.0	18,259.0
**Union**	2592,790.0	650,952.0	9434,406.0	1118,489.0	1574,191.0	909,295.0
**Shingkong**	2609,941.0	1368,802.0	13,921,464.0	811,343.0	3609,236.0	223,047.0
**South China**	1396,002.0	988,888.0	7396,396.0	465,509.0	1401,200.0	332,283.0
**Cathay Century**	2184,944.0	651,063.0	10,422,297.0	749,893.0	3355,197.0	555,482.0
**Allianz President**	1211,716.0	415,071.0	5606,013.0	402,881.0	854,054.0	197,947.0
**Newa**	1453,797.0	1085,019.0	7695,461.0	342,489.0	3144,484.0	371,984.0
**AIU**	757,515.0	547,997.0	3631,484.0	995,620.0	692,731.0	163,927.0
**North America**	159,422.0	182,338.0	1141,951.0	483,291.0	519,121.0	46,857.0
**Federal**	145,442.0	53,518.0	316,829.0	131,920.0	355,624.0	26,537.0
**Royal & Sunalliance**	84,171.0	26,224.0	225,888.0	40,542.0	51,950.0	6491.0
**Aisa**	15,993.0	10,502.0	52,063.0	14,574.0	82,141.0	4181.0
**AXA**	54,693.0	28,408.0	245,910.0	49,864.0	0.1	18,980.0
**Mitsui Sumitomo**	163,297.0	235,094.0	476,419.0	644,816.0	142,370.0	16,976.0
**Mean**	1544,214.6	828,963.1	7832,893.0	667,964.3	1602,872.9	477,732.8

**Note:** Units in NT$ thousands.

Efficiencies of 24 non-life insurance companies are analysed by proposed reconciled multiplicative relational (RMR) model. In Table 2 the efficiency values after the first and the second steps are presented. Maximum relational efficiency values of the DMUs found in the first step, and the final efficiencies after the reconciliation step is presented.

**Table 2:** Efficiency scores of RMR model after the first and the second steps.

	**Step 1**			**Step 2**		
**Company**	***E_max_***	***E^1^_max_***	***E^2^_max_***	***E***	***E^1^***	***E^2^***
**Taiwan Fire**	0.699	0.993	0.713	0.699	**0.992**	**0.705**
**Chung Kuo**	0.625	0.998	0.626	0.625	0.998	0.626
**TaiPing**	0.690	0.690	1.000	0.690	0.690	1.000
**China Mariners**	0.304	0.724	0.432	0.304	0.724	**0.420**
**Fubon**	0.767	0.838	1.000	**0.757**	**0.799**	**0.947**
**Zurich**	0.390	0.964	0.406	**0.389**	**0.961**	**0.405**
**Taian**	0.277	0.752	0.538	0.277	**0.671**	**0.412**
**Ming Tai**	0.275	0.726	0.511	0.275	**0.663**	**0.415**
**Central**	0.223	1.000	0.292	0.223	**0.990**	**0.225**
**The First**	0.466	0.862	0.674	**0.457**	**0.835**	**0.547**
**Kuo Hua**	0.164	0.741	0.327	**0.158**	**0.685**	**0.231**
**Union**	0.760	1.000	0.760	0.760	1.000	0.760
**Shingkong**	0.208	0.811	0.543	**0.207**	**0.681**	**0.304**
**South China**	0.289	0.725	0.518	0.289	**0.670**	**0.431**
**Cathay Century**	0.614	1.000	0.705	**0.606**	**0.972**	**0.624**
**Allianz President**	0.320	0.907	0.385	0.320	**0.886**	**0.362**
**Newa**	0.360	0.723	1.000	**0.330**	**0.481**	**0.686**
**AIU**	0.259	0.794	0.374	**0.258**	**0.781**	**0.330**
**North America**	0.411	1.000	0.416	0.411	1000	**0.411**
**Federal**	0.547	0.933	0.901	**0.535**	**0.816**	**0.656**
**Royal & Sunalliance**	0.201	0.751	0.280	**0.199**	**0.742**	**0.268**
**Aisa**	0.590	0.590	1.000	0.590	0.590	1.000
**AXA**	0.420	0.850	0.560	**0.418**	**0.832**	**0.502**
**Mitsui Sumitomo**	0.135	1.000	0.335	**0.094**	**0.942**	**0.099**

In Table 2, it can be seen that three companies’ maximum relational efficiencies of all DMUs found in step 1 are already multiplicatively-consistent and do not change in the reconciliation step. Taiping, Union and Aisa are such companies. On the other hand, other companies’ at least one DMU efficiency is changed in reconciliation step. All three DMU efficiencies (including main and sub-DMU efficiencies) of Fubon, Zurich, The First, Kuo Hua, Shingkong, Cathay Century, Newa, AIU, Federal, Royal & Sunalliance, AXA and Mitsui Sumitomo are reconciled and changed in the second step.

Results of the proposed model are compared with the independent model in [22], and the multiplicative relational (MR) model presented in [1]. The independent model omits the interconnections between main and sub-DMUs. Hence, it assumes that there are three different and independent DMUs, and each DMU’s efficiency is optimized independently with simple CRS (Constant returns to scale) model presented in [23]. Multiplicative relational model is the same model presented in [1], which prioritized the second sub-DMU over the first one. In Table 3, the resulting efficiency values of independent, MR and RMR models are presented.

**Table 3:** Efficiency scores of independent, MR and RMR models for main process (*E*), first stage (*E^1^*) and second stage (*E^2^*).

**Company**	**Independent**			**MR**			**RMR**		
	***E***	***E^1^***	***E^2^***	***E***	***E^1^***	***E^2^***	***E***	***E^1^***	***E^2^***
**Taiwan Fire**	0.984	0.993	0.713	0.699	0.993	0.704	0.699	0.992	0.705
**Chung Kuo**	**1.000**	0.998	0.626	0.625	0.998	0.626	0.625	0.998	0.626
**TaiPing**	0.988	0.690	**1.000**	0.690	0.690	**1.000**	0.690	0.690	**1.000**
**China Mariners**	0.488	0.724	0.432	0.304	0.724	0.420	0.304	0.724	0.420
**Fubon**	**1.000**	0.838	**1.000**	0.767	0.831	0.923	0.757	0.799	0.947
**Zurich**	0.594	0.964	0.406	0.390	0.961	0.406	0.389	0.961	0.405
**Taian**	0.470	0.752	0.538	0.277	0.671	0.412	0.277	0.671	0.412
**Ming Tai**	0.415	0.726	0.511	0.275	0.663	0.415	0.275	0.663	0.415
**Central**	0.327	**1.000**	0.292	0.223	**1.000**	0.223	0.223	0.990	0.225
**The First**	0.781	0.862	0.674	0.466	0.862	0.541	0.457	0.835	0.547
**Kuo Hua**	0.283	0.741	0.327	0.164	0.647	0.253	0.158	0.685	0.231
**Union**	**1.000**	**1.000**	0.760	0.760	**1.000**	0.760	0.760	**1.000**	0.760
**Shingkong**	0.353	0.811	0.543	0.208	0.672	0.309	0.207	0.681	0.304
**South China**	0.470	0.725	0.518	0.289	0.670	0.431	0.289	0.670	0.431
**Cathay Century**	0.979	**1.000**	0.705	0.614	**1.000**	0.614	0.606	0.972	0.624
**Allianz President**	0.472	0.907	0.385	0.320	0.886	0.362	0.320	0.886	0.362
**Newa**	0.635	0.723	**1.000**	0.360	0.628	0.574	0.330	0.481	0.686
**AIU**	0.427	0.794	0.374	0.259	0.794	0.326	0.258	0.781	0.330
**North America**	0.822	**1.000**	0.416	0.411	**1.000**	0.411	0.411	**1.000**	0.411
**Federal**	0.935	0.933	0.901	0.547	0.933	0.586	0.535	0.816	0.656
**Royal & Sunalliance**	0.333	0.751	0.280	0.201	0.732	0.274	0.199	0.742	0.268
**Aisa**	**1.000**	0.590	**1.000**	0.590	0.590	**1.000**	0.590	0.590	**1.000**
**AXA**	0.599	0.851	0.560	0.420	0.843	0.499	0.418	0.832	0.502
**Mitsui Sumitomo**	0.257	**1.000**	0.335	0.135	0.429	0.314	0.094	0.942	0.099

According to independent model, 4 main processes (Chung Kuo, Fubon, Union and Aisa) are efficient, whereas to MR and RMR models there is not any efficient main process. Independent model indicates that the first stages of 5 companies (Central, Union, Cathay Century, North America and Mitsui Sumitomo) are efficient. However, MR model results show that only four of them is efficient (leaving Mitsui Sumitomo out). According to proposed RMR model, the first stages of Union and North America are efficient. Similar results are found for the efficiency of the second stages. Independent model favors 4 companies’ second stages as efficient (TaiPing, Fubon, Newa and Aisa). According to RM model only two of them (TaiPing and Aisa) are efficient, which is the same as RMR model. It can be seen that, MR model has fewer efficient nodes than independent model, while RMR model has less than MR has.

In Table 4, the rank scores of the companies for overall (R) and sub-processes (*R*^1^, *R*^2^) are presented. If there are >1 efficient node, the rank values of those nodes are calculated as in [1]. For n efficient DMU, rank value of each of them is: $r=\frac{1+\ldots +n}{n}$.

**Table 4:** Efficiency ranking scores of independent, MR and RMR models for main process (*R*), first stage (*R*^1^) and second stage (*R*^2^).

**Company**	**Independent**			**MR**			**RMR**		
	***R***	***R*^1^**	***R*^2^**	***R***	***R*^1^**	***R*^2^**	***R***	***R*^1^**	***R*^2^**
**Taiwan Fire**	6	7	7	3	6	5	3	4	5
**Chung Kuo**	2.5	6	10	5	5	6	5	3	8
**TaiPing**	5	23	2.5	4	16	1.5	4	17	1.5
**China Mariners**	14	21	16	15	15	13	15	16	13
**Fubon**	2.5	13	2.5	1	12	3	2	13	3
**Zurich**	13	8	18	12	7	17	12	7	17
**Taian**	16	16	13	17	18	15	17	20	15
**Ming Tai**	19	19	15	18	20	14	18	22	14
**Central**	22	3	23	20	2.5	24	20	5	23
**The First**	10	11	9	9	10	10	9	10	10
**Kuo Hua**	23	18	22	23	21	23	23	18	22
**Union**	2.5	3	6	2	2.5	4	1	1.5	4
**Shingkong**	20	14	12	21	17	21	21	19	20
**South China**	16	20	14	16	19	12	16	21	12
**Cathay Century**	7	3	8	6	2.5	7	6	6	9
**Allianz President**	15	10	19	14	9	18	14	9	18
**Newa**	11	22	2.5	13	22	9	13	24	6
**AIU**	18	15	20	19	13	19	19	14	19
**North America**	9	3	17	11	2.5	16	11	1.5	16
**Federal**	8	9	5	8	8	8	8	12	7
**Royal & Sunalliance**	21	17	24	22	14	22	22	15	21
**Aisa**	2.5	24	2.5	7	23	1.5	7	23	1.5
**AXA**	12	12	11	10	11	11	10	11	11
**Mitsui Sumitomo**	24	3	21	24	24	20	24	8	24

To be able to compare the rankings, Spearman’s rank correlation coefficient (*ρ*) is used. In Table 5 rank correlations of main and sub-DMUs between compared models are presented. The ranking of the overall efficiencies (*R*) with reconciled and relational model is almost identical, except for the first and the second ranks. Difference is the reconciliation process of the Fubon. After the reconciliation, main process efficiency decreases from 0.767 to 0.757, which changes Fubon’s ranking to second. On the other hand, ranking values of independent model is more diverse than relational and reconciled models. For example, first stage ranking of Mitsui Sumitomo is 20 according to conventional MR model, and increases to number 8 with RMR model. This situation is not unexpected since its maximum relational efficiency value is 1 according to first step of RMR model.

**Table 5:** Spearman’s rank correlation coefficient (*ρ*) values between models.

	**Main-DMU**			**Sub-DMU 1**			**Sub-DMU 2**		
**Models**	**Ind.**	**MR**	**RMR**	**Ind.**	**MR**	**RMR**	**Ind.**	**MR**	**RMR**
**Ind.**	1.000	0.972	0.972	1.000	0.749	0.915	1.000	0.917	0.940
**MR**		1.000	0.999		1.000	0.854		1.000	0.983
**RMR**			1.000			1.000			1.000

The tabulated critical value of Spearman’s rank correlation coefficient for *n* = 24 and α=0.05 (α is the level of significance) is 0.344 [24]. Therefore, all three model rankings are highly correlated. There is a very high rank correlation of 0.999 between proposed and MR model, while a lesser correlation of 0.972 between independent and other two models. These results show that proposed model gives close results to relational model than independent model on the main process efficiency side. However, sub-DMU efficiency rankings show different characteristics. For the first stage efficiency rankings, reconciled model is closer to independent model with a rank correlation coefficient of 0.915, which is distinctly higher than the correlation of 0.854 between MR and RMR model. Conversely, reconciled model rankings are closer to relational model for second stage efficiencies with a very high value of 0.983, while rank correlation between independent and RMR model is 0.940. The rank correlation scores found show that, the direction of the ranks determined by the proposed model is the same with other two models and the proposed model is an equally valid model with compared to two other models [6,18].

### *Chinese bank branches*

In this section, proposed RMR model is applied to determine the efficiency decompositions of 10 bank branches of China Construction Bank (CCB) in Anhui province, PR China. The dataset is for year 2004, and used in several studies for efficiency analysis [3,6,25]. The process of bank branches is modelled as a two-stage system. In the first stage, the inputs are number of employees (X1), fixed assets (X2), and expenses (X3); where the outputs are credit (Z1) and interbank loans (Z2). In the second stage, the outputs from the first stage (Z1 and Z2) are used as inputs to generate two outputs, as loan (Y1) and profit (Y2). Dataset is presented in Table 6.

**Table 6:** Dataset for 10 bank branches of Chinese Construction Bank [3].

**Bank Branch**	**X1 (10^3^)**	**X2 (10^8^ ¥)**	**X3 (10^8^ ¥)**	**Z1 (10^8^ ¥)**	**Z2 (10^8^ ¥)**	**Y1 (10^8^ ¥)**	**Y2 (10^8^ ¥)**
**Maanshan**	0.526	0.478	0.385	49.917	5.461	34.990	0.843
**Anqing**	0.713	1.236	0.555	37.495	4.083	20.601	0.486
**Huangshan**	0.443	0.446	0.342	20.985	0.690	8.633	0.129
**Fuyang**	0.638	1.248	0.457	45.051	1.724	9.235	0.302
**Suzhou**	0.575	0.705	0.404	38.162	2.249	12.017	0.314
**Chuzhou**	0.432	0.645	0.401	30.168	2.335	13.813	0.377
**Luan**	0.510	0.724	0.371	26.539	1.342	5.096	0.145
**Chizhou**	0.322	0.336	0.233	16.124	0.489	5.980	0.093
**Chaohu**	0.423	0.668	0.347	22.185	1.177	9.235	0.200
**Bozhou**	0.256	0.342	0.159	13.436	0.406	2.533	0.006
**Mean**	0.484	0.683	0.365	30.006	1.996	12.213	0.290

Efficiencies of 10 bank branches are calculated with the proposed RMR model. In Table 7, the efficiency values after the first and the second steps of the proposed model are presented.

**Table 7:** Efficiency scores of RMR model after the first and the second steps.

	**Step 1**			**Step 2**		
**Bank Branch**	***E_max_***	***E^1^_max_***	***E^2^_max_***	***E***	***E^1^***	***E^2^***
**Maanshan**	1.000	1.000	1.000	1.000	1.000	1.000
**Anqing**	0.434	0.553	0.785	0.434	0.553	0.785
**Huangshan**	0.293	0.499	1.000	0.293	**0.312**	**0.938**
**Fuyang**	0.301	0.759	0.970	0.301	**0.383**	**0.787**
**Suzhou**	0.355	0.729	0.834	0.355	**0.526**	**0.675**
**Chuzhou**	0.545	0.736	1.000	0.545	**0.599**	**0.910**
**Luan**	0.179	0.552	0.632	0.179	**0.363**	**0.493**
**Chizhou**	0.282	0.533	1.000	0.282	**0.305**	**0.923**
**Chaohu**	0.328	0.553	1.000	0.324	**0.374**	**0.867**
**Bozhou**	0.175	0.650	0.498	0.175	**0.546**	**0.320**

It can be seen from Table 7 that, Maanshan and Anqing branches’ maximum efficiency values are already multiplicatively-consistent, and do not change in the second step. On the other hand, all other companies’ sub-DMU efficiencies are changed and reduced after the reconciliation process. Main DMU efficiencies stay the same except Chaohu branch, which is reconciled in the second step to a lesser value. Compared efficiency results of the proposed, independent and MR models are presented in Table 8. Note that, MR model is the same model presented in [1], which prioritized the second sub-DMU over the first one.

**Table 8:** Efficiency scores of independent, MR and RMR models for main process (*E*), first stage (*E^1^*) and second stage (*E^2^*).

**Bank Branch**	**Independent**			**MR**			**RMR**		
	***E***	***E^1^***	***E^2^***	***E***	***E^1^***	***E^2^***	***E***	***E^1^***	***E^2^***
**Maanshan**	**1.000**	**1.000**	**1.000**	**1.000**	**1.000**	**1.000**	**1.000**	**1.000**	**1.000**
**Anqing**	0.434	0.554	0.786	0.434	0.553	0.786	0.434	0.553	0.785
**Huangshan**	0.293	0.499	**1.000**	0.293	0.293	**1.000**	0.293	0.312	0.938
**Fuyang**	0.301	0.759	0.970	0.301	0.321	0.939	0.301	0.383	0.787
**Suzhou**	0.355	0.729	0.833	0.355	0.430	0.825	0.355	0.526	0.675
**Chuzhou**	0.545	0.736	**1.000**	0.545	0.545	**1.000**	0.545	0.599	0.910
**Luan**	0.179	0.552	0.632	0.179	0.288	0.621	0.179	0.363	0.493
**Chizhou**	0.282	0.533	**1.000**	0.282	0.305	0.923	0.282	0.305	0.923
**Chaohu**	0.328	0.553	**1.000**	0.328	0.385	0.854	0.324	0.374	0.867
**Bozhou**	0.175	0.650	0.498	0.175	0.372	0.470	0.175	0.546	0.320

All three models compared favour only Maanshan branch’s main DMU as efficient. Similarly, only Maanshan branch first-stage is efficient according to all models. Independent model assesses second-stages of 5 branches as efficient (Maanshan, Huangshan, Chuzhou, Chizhou and Chaohu), while according to MR model only 3 of them (Maanshan, Huangshan and Chuzhou) is efficient. However, proposed RMR model discriminatively selects only one second-stage (Maanshan) as an efficient process. Results show that, MR model has fewer or equal number of efficient nodes than independent model, while RMR model has less than or equal to which MR has. The rank scores of the bank branches are presented in Table 9 for overall (*R*) and sub-processes (*R*^1^, *R*^2^). Also in Table 10, rank correlations of the main and sub-DMUs between compared models are presented.

**Table 9:** Efficiency ranking scores of independent, MR and RMR models for main process (*R*), first stage (*R*^1^) and second stage (*R*^2^).

**Company**	**Independent**			**MR**			**RMR**		
	***R***	***R*^1^**	***R*^2^**	***R***	***R*^1^**	***R*^2^**	***R***	***R*^1^**	***R*^2^**
**Maanshan**	1	1	3	1	1	2	1	1	1
**Anqing**	3	6	8	3	2	8	3	3	7
**Huangshan**	7	10	1	7	9	2	7	9	2
**Fuyang**	6	2	6	6	7	4	6	6	6
**Suzhou**	4	4	7	4	4	7	4	5	8
**Chuzhou**	2	3	4	2	3	2	2	2	4
**Luan**	9	8	9	9	10	9	9	8	9
**Chizhou**	8	9	2	8	8	5	8	10	3
**Chaohu**	5	7	5	5	5	6	5	7	5
**Bozhou**	10	5	10	10	6	10	10	4	10

**Table 10:** Spearman’s rank correlation coefficient (*ρ*) values between models.

	**Main-DMU**			**Sub-DMU 1**			**Sub-DMU 2**		
**Models**	**Ind.**	**MR**	**RMR**	**Ind.**	**MR**	**RMR**	**Ind.**	MR	**RMR**
**Ind.**	1.000	1.000	1.000	1.000	0.685	0.818	1.000	0.877	0.952
**MR**		1.000	1.000		1.000	0.879		1.000	0.902
**RMR**			1.000			1.000			1.000

The rankings of the overall efficiencies (*R*) of all compared models are the same. However, there are differences in the sub-stage rankings. RMR model ranking is closer to MR ranking in the first sub-stage, while is closer to independent model in the second sub-stage. The tabulated critical value of Spearman’s rank correlation coefficient for *n* = 10 and α=0.05 is 0.552 [13]. All three model rankings are highly correlated in all main and sub-stages. Proposed RMR model ranking is more correlated to MR model in the first sub-stage with *ρ* = 0.879, and independent model in the second sub-stage with *ρ* = 0.952. Obtained results show that the direction of the ranks of the proposed model is the same with other two models and proposed RMR model is a valid model as two other compared models.

Considering the results of the applications of both Taiwanese non-life insurance companies and Chinese bank branches, it can be said that some results are varies and dataset specific. In the previous application with Taiwanese non-life insurance companies’ dataset, the proposed RMR model results are closer to conventional MR model than the independent model in Main-DMU and Sub-DMU 2. Conversely, in the second example with Chinese bank branches, RMR model rankings are closer to MR model in Sub-DMU 1. On the other hand, all three models’ results have the same direction of rankings and highly close for both applications.

## **Limitations**

Presented model does not prioritize any DMU over another, and aims a fairer efficiency assessment. Therefore, if the main-DMU or any sub-DMU has more importance than others do, presented model is not suitable to use. Additionally, proposed RMR model consists more optimization model (3 linear and 1 quadratic) than the original model [1] (2 linear). Depending on the software used, this may result less computational efficiency, which might be significant in large-scale datasets [26]. Finally, presented RMR model is possible only under the CRS model assumption as the original relational model [1,27]. Expanding the model assumption to variable returns to scale (VRS) might be another area of a future study.

## Ethics statements

The method used in the study did not involve any human or animal subjects.

## CRediT author statement

**M. Burak Erturan:** Conceptualization, Methodology, Validation, Formal analysis, Writing – Original Draft, Writing – Review & Editing Visualization.

## Declaration of competing interest

The authors declare that they have no known competing financial interests or personal relationships that could have appeared to influence the work reported in this paper.

## Data Availability

Data is presented within the article
